# Protective effect of exogenous matrix metalloproteinase-9 on chronic renal failure

**DOI:** 10.3892/etm.2013.1409

**Published:** 2013-11-15

**Authors:** LEI WANG, JUE WANG, YONG WANG, QIANG FU, YONG-HUA LEI, ZHI-YONG NIE, JIANXIN QIU, TING-YI BAO

**Affiliations:** 1 Department of Urologic Surgery, Institute of Infectious Diseases, Tangdu Hospital, Fourth Military Medical University, Xi’an, Shaanxi 710038, P.R. China; 2 Department of Urologic Surgery, Tangdu Hospital, Fourth Military Medical University, Xi’an, Shaanxi 710038, P.R. China; 3 Department of Urologic Surgery, Tenth Hospital of PLA, Wuwei, Gansu 733000, P.R. China

**Keywords:** adenine, chronic renal failure, MMP-9, TIMP-1

## Abstract

Matrix metalloproteinases (MMPs) and tissue inhibitors of metalloproteinases (TIMPs) have pivotal functions in extracellular matrix turnover and are involved in chronic kidney diseases. However, the exact functions of MMPs in chronic renal failure (CRF) have yet to be demonstrated. The aim of the present study was to examine the effects of MMP-9 on CRF. An adenine-induced model of CRF was generated in rabbits. Following the injection of MMP-9 into the renal arteries of the rabbits, significant improvements in renal morphology and serum levels of creatinine and urea nitrogen were observed. Furthermore, MMP-9 administration was shown to decrease the serum TIMP-1 concentration and upregulate renal MMP-9 expression. These results demonstrate a directly protective role for MMP-9 in CRF.

## Introduction

Chronic renal failure (CRF) is characterized by progressive tubulointerstitial fibrosis and glomerulosclerosis (1), which are associated with the accumulation of extracellular matrix (ECM) proteins. The major physiological regulators of ECM are matrix metalloproteinases (MMPs). MMPs belong to a large family of proteolytic enzymes that degrade various types of ECM components, such as collagens, laminin, fibronectin, vitronectin, aggrecan, enactin, tenascin, elastin and proteoglycans, and a number of non-ECM substrates, including signaling molecules and cell adhesion molecules (2,3).

To date, six groups of MMPs have been identified based on substrate and sequence homology. These are collagenases, gelatinases, stromelysins, matrilysins, membrane-type MMPs and other MMPs. MMP-9 is a gelatinase and cleaves denatured collagens (gelatins) and laminin, as well as certain chemokines (2). MMP-9, like other MMPs, is expressed in the kidney in a variety of vertebrates, including rabbits, and appears to be mainly confined to the glomerulus (4). MMP-9 is also expressed in the collecting duct of rabbits (5).

Due to their ability to degrade the structure of most tissues, MMPs are responsible for the accumulation of ECM proteins, which may lead to a fibrotic state in various organs, including the kidney, when MMP activity and expression levels decrease. The expression and activity of MMPs are tightly regulated at multiple levels, including gene transcription, post-transcriptional modification and, in particular, the interaction with circulating inhibitors that serve to limit the activity of the MMPs (6). Tissue inhibitors of metalloproteinases (TIMPs) are endogenous, specific inhibitors of MMPs. TIMPs non-covalently bind to MMPs to form high-affinity complexes and block the binding of MMPs to their substrates (7). Four TIMPs (TIMP-1-4) have been identified in vertebrates. TIMP-1 is capable of inhibiting the activity of most MMPs, with the exceptions of MMP-14, 16 and 24 (8), and is important in maintaining the balance between ECM deposition and degradation. TIMP-1 expression has been observed in the glomeruli of rats and humans (9,10).

Accumulating data have shown that MMP-9 and TIMP-1 function differentially in chronic kidney diseases (CKDs). The downregulation of MMPs or upregulation of TIMPs leads to an imbalance in the MMP/TIMP ratio, which results in ECM accumulation (1). This may promote CKD progression. Studies of various kidney disease models and patients with CKD have demonstrated a reduction in the activity and expression of MMP-9, in contrast with an increase in TIMP-1 expression (11,12). However, in diabetic nephropathy, a marked increase in the MMP-9/TIMP-1 ratio has been identified (13). The upregulation of MMP-9 has also been observed in diabetic CKD arteries, which was noted to be associated with arterial stiffening, impaired angiogenesis and endothelial dysfunction (14).

As a result of the contradictory observations regarding MMP-9, in the present study the effects of MMP-9 on CRF were examined through the injection of MMP-9 into rabbit renal arteries in an adenine-induced model of CRF. Adenine-induced CRF has been demonstrated to be accompanied by an increased expression of inflammatory mediators, including cyclooxygenase-2 (COX-2) (15); thus, the expression of COX-2, as well as TIMP-1, was investigated in this study.

## Materials and methods

### Reagents

Adenine (A8626) was purchased from Sigma (St. Louis, MO, USA). Anti-MMP-9 (sc-21733), anti-TIMP-1 (sc-21734), anti-COX-2 (sc-376861) and anti-β-actin (sc-47778) antibodies were obtained from Santa Cruz Biotechnology, Inc. (Santa Cruz, CA, USA). The horseradish peroxidase-conjugated secondary antibody and streptavidin-biotin complex (sABC) kits used in the study were purchased from Wuhan Boster Biological Technology Co. Ltd., (Wuhan, China), while the kits for serum creatinine (SCr) and blood urea nitrogen (BUN) were purchased from Nanjing Jiancheng Technology Company, Ltd. (Nanjing, China). MMP-9 protein (911-MP-010, purity >90%) and enzyme-linked immunosorbent assay (ELISA) kits for MMP-9 (DMP900) and TIMP-1 (DTM100) were purchased from R&D Systems (Minneapolis, MN, USA). TRIzol^®^ reagent (15596-026) and SYBR^®^-GreenER™ qPCR SuperMix Universal kit (11762-500) were purchased from Invitrogen Life Technologies (Carlsbad, CA, USA). Moloney murine leukemia virus (M-MLV) reverse transcriptase was purchased from Promega Corp. (Madison, WI, USA).

### Animals

All animal use procedures were in strict accordance with National Institutes of Health guidelines and were approved by the Fourth Military Medical University (Xi’an, China). Male rabbits (weight, 2.0–2.5 kg; age, 4–5 months) were provided by the Animal Center of the Fourth Military Medical University. The rabbits were maintained in separate cages at a constant humidity and temperature, with food and water available *ad libitum*. The animal room was on a 12/12-h light/dark cycle. The 30 male rabbits were randomly divided into control, CRF and MMP-9 groups. The rabbits in the CRF and MMP groups were treated with adenine (350 mg/kg body weight) once a day by oral gavage for a total of 10 weeks. The control rabbits were treated with an equal volume of vehicle. At the 10th week following the adenine administration, the rabbits in the MMP-9 group were anesthetized with a mixture of diazepam, haloperidol and dihydroetorphine, and the bilateral renal arteries were exposed. MMP-9 was subsequently injected into the bilateral renal arteries (1 μg each artery). The rabbits in the control and CRF groups underwent an identical surgical procedure and were injected with an equal volume of vehicle. The surgery was strictly conducted under sterile conditions. All rabbits were treated with 4×10^5^ units penicillin following the surgery, and were kept in a room with specific pathogen free (SPF) standards.

### Measurements of MMP-9, TIMP-1, SCr, BUN and proteinuria

The levels of SCr and BUN were measured with the picric acid and Urease-Berthelot methods, respectively, in accordance with the kit manufacturers’ instructions. The serum levels of MMP-9 and TIMP-1 were measured using ELISA, according to the recommended instructions. Proteinuria from sporadic urea was measured with a regular medical examination method.

### Kidney section and protein lysate preparation

All rabbits were sacrificed at the 14th week (the fourth week subsequent to MMP-9 injection) and the bilateral kidneys were removed. A sample of renal tissue was fixed in paraffin and cut into 5-μm-thick sections. The sections were used for hematoxylin and eosin (H&E) staining to examine the renal morphology. An additional sample of renal tissue was lysed in radioimmunoprecipitation assay (RIPA) buffer and total protein was extracted for immunoblotting, while a third sample was used for mRNA extraction.

### Quantitative reverse transcription-polymerase chain reaction (qPCR)

qPCR was performed in order to evaluate the mRNA expression of MMP-9, TIMP-1 and COX-2. Total RNA was extracted from the renal tissues using TRIzol reagent, in accordance with the manufacturer’s instructions, and the RNA was reverse-transcribed into cDNA using M-MLV reverse transcriptase. qPCR was performed using a SYBR-GreenER qPCR SuperMix Universal kit with an ABI StepOnePlus™ real-time PCR system (Applied Biosystems; Life Technologies, Carlsbad, CA, USA). The following cycling profile was used subsequent to an initial denaturation at 95ºC for 5 min: denaturation at 95ºC for 30 sec, annealing at 60ºC for 30 sec and extension at 72ºC for 45 sec. Amplification was performed for 39 cycles. The primers specific for the examined genes are shown in Table I. The results are presented as the levels of expression relative to those of the controls subsequent to normalizing to β-actin using the 2^−ΔΔCt^ method.

### Western blot analysis

The protein levels of MMP-9, TIMP-1 and COX-2 in the kidneys were assessed with western blotting, in accordance with a previous study (16). Whole tissue proteins were separated electrophoretically in 4–12% sodium dodecyl sulfate-polyacrylamide gel electrophoresis (SDS-PAGE) gels, prior to being transferred to nitrocellulose membranes. Following 30 min of blocking with 2.5% non-fat milk, the membranes were incubated with primary antibodies (1:2,000) at 4ºC overnight, prior to 1 h incubation with horseradish peroxidase-conjugated secondary antibody (1:2,000). The membranes were adequately washed with phosphate-buffered saline (PBS) containing 0.5% Tween 20 subsequent to each treatment with antibody. The membranes were developed with Amersham ECL Western Blotting Analysis system (Cat. No.: RPN2109, GE healthcare, Chalfont St Giles, UK) and then exposed to X-ray film. The protein levels of MMP-9, TIMP-1 and COX-2 are expressed as the ratio of the band optical intensity to that of β-actin.

### Statistical analysis

Data are presented as the mean ± standard error of the mean. The statistical analysis was performed using SPSS 13 statistical software (SPSS, Inc., Chicago, IL, USA) and a Dunnett’s test was utilized for multiple comparisons. P<0.05 was considered to indicate a statistically significant difference.

## Results

### Induction of CRF by adenine

**Figure 1:**
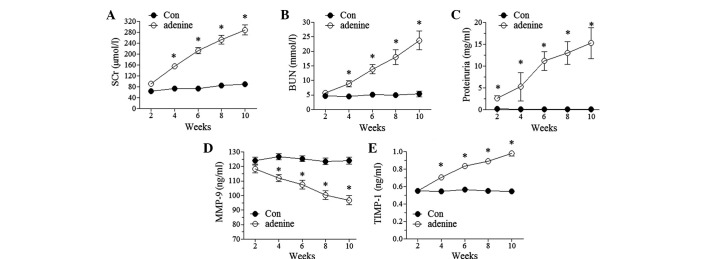
Changes in the levels of serum creatinine (SCr), blood urea nitrogen (BUN) and proteinuria and the serum levels of matrix metalloproteinase-9 (MMP-9) and tissue inhibitor of metalloproteinase-1 (TIMP-1) in adenine-induced chronic renal failure (CRF). The rabbits in the CRF and MMP-9 groups were treated with adenine daily by oral gavage to induce CRF (adenine, n=20). The control rabbits (Con, n=10) were treated with an equal volume of water. Serum and sporadic urea were collected prior to adenine treatment (week 0) and every two weeks during the adenine treatment, in order to monitor the levels of: (A) SCr, (B) BUN, (D) MMP-9 and (E) TIMP-1 and (C) the urea level of proteinuria. ^*^P<0.05 vs. Con group.

Adenine administration has been frequently used to induce CRF models in various animals, including mice and rats (15,17). In this study, the rabbits that were treated with adenine exhibited a CRF-like change. The levels of SCr and BUN were observed to significantly increase at the fourth week following adenine administration (P<0.05 versus the control) and the levels continually increased with time during the total period of adenine administration (Fig. 1A and B). Proteinuria was apparent early in the second week subsequent to adenine administration and was also observed to increase with time during the total period of adenine administration (Fig. 1C). These data indicated the presence of glomerular damage. By contrast, there were no changes in the levels of SCr and BUN in the control group and no proteinuria. These results were suggestive of the successful induction of CRF. The increased levels of SCr, BUN and proteinuria induced by adenine administration remained unchanged up to four weeks subsequent to the cessation of adenine administration in this study (data not shown).

### Serum levels of MMP-9 and TIMP-1 in the CRF model

Experiments were conducted to examine whether CRF affected the serum levels of MMP-9 and TIMP-1. The results showed that the levels of MMP-9 decreased and TIMP-1 increased in a time-dependent manner in the adenine-treated rabbits, compared with virtually no change in the control rabbits (Fig. 1D and E). The altered levels of MMP-9 and TIMP-1 remained unchanged up to four weeks subsequent to the cessation of adenine administration (data not shown).

### Exogenous MMP-9 improves renal function and morphology

**Figure 2:**
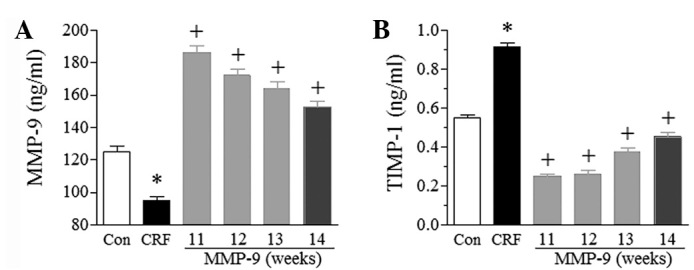
Effect of matrix metalloproteinase-9 (MMP-9) on the serum levels of MMP-9 and tissue inhibitor of metalloproteinase-1 (TIMP-1). Rabbits in the MMP-9 group (n=10) were injected with MMP-9 at the 10th week. Rabbits in the control (Con; n=10) and chronic renal failure (CRF; n=10) groups were injected with an equal volume of water. Serum was collected every week to monitor the serum levels of MMP-9 and TIMP-1. There were no significant changes in the serum levels of MMP-9 and TIMP-1 in the control and CRF groups following MMP-9 treatment. The data presented in this figure are from the 11th week. ^*^P<0.05 vs. Con group; ^+^P<0.05 vs. CRF group.

To confirm whether MMP-9 was involved in CRF, MMP-9 was injected into the bilateral renal arteries. The treatment was demonstrated to significantly increase the serum level of MMP-9 (P<0.05). As shown in Fig. 2A, at the 11th week (the first week following MMP-9 injection), the serum level of MMP-9 was two-fold higher in the MMP-9 group than in the CRF group, and more than two-fold higher than that in the control group. The elevated serum level of MMP-9 in the MMP-9 group gradually decreased; however, it remained higher than the levels in the control and CRF groups throughout the total experimental period of the study.

**Figure 3:**
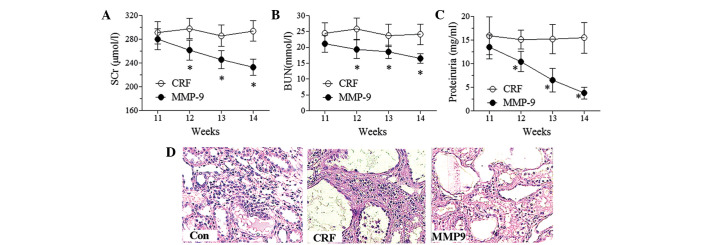
Effect of matrix metalloproteinase-9 (MMP-9) on serum creatinine (SCr), blood urea nitrogen (BUN), proteinuria and renal morphology. Rabbits in the MMP-9 group (n=10) were injected with MMP-9 at the 10th week. Rabbits in the control (Con; n=10) and chronic renal failure (CRF; n=10) groups were injected with an equal volume of water. Serum and sporadic urea were collected every week to monitor the levels of: (A) SCr, (B) BUN and (C) the urea level of proteinuria. (D) At the 14th week (the fourth week subsequent to the MMP-9 treatment), the animals were sacrificed and the kidneys were removed for hematoxylin and eosin (H&E) staining to examine the morphological changes (magnification, ×100). ^*^P<0.05 vs. CRF group.

The levels of SCr, BUN and proteinuria were measured to evaluate the effect of MMP-9 on renal function. MMP-9 treatment time-dependently decreased the levels of SCr, BUN and proteinuria (Fig. 3A–C). Significant effects appeared early at the second week subsequent to MMP-9 treatment (P<0.05). By contrast, no significant changes were observed in the levels of SCr, BUN and proteinuria in the CRF group during the experimental period. These results demonstrated the protective effect of MMP-9 on renal function. MMP-9 is an ECM proteolytic enzyme. The accumulation of ECM is an important pathological feature in the development of glomerulosclerosis and tubulointerstitial fibrosis. Thus, renal morphology was examined to further investigate the effect of MMP-9 (Fig. 3D). H&E staining showed no abnormal morphological changes in the control group. However, in the CRF group, a number of glomeruli were observed to have developed focal segmental glomerulosclerosis, basement membrane thickening, mesangial cell proliferation with ECM expansion, fibrin accumulation in the renal capsule, tubulointerstitial fibrosis and tubular basement membrane thickening. MMP-9 treatment decreased the glomerular lesions and, more prominently, largely prevented fibrin accumulation in the renal capsule and reduced the development of tubulointerstitial thickness.

### Exogenous MMP-9 increases the renal expression of MMP-9

**Figure 4:**
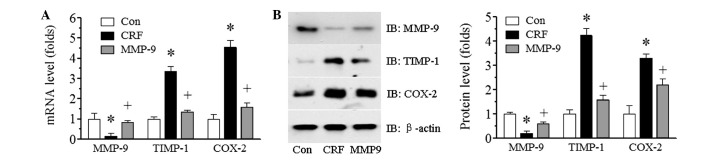
Effect of matrix metalloproteinase-9 (MMP-9) on the tissue expression of MMP-9, tissue inhibitor of metalloproteinase-1 (TIMP-1) and cyclooxygenase-2 (COX-2). Rabbits in the MMP-9 group (n=10) were injected with MMP-9 at the 10th week. Rabbits in the control (Con; n=10) and chronic renal failure (CRF; n=10) groups were were injected with an equal volume of water. At the 14th week (the fourth week subsequent to the MMP-9 treatment), the animals were sacrificed and the kidneys were removed. Samples of kidney tissue were obtained and used as follows: (A) qPCR to examine the mRNA expression of MMP-9, TIMP-1 and COX-2; and (B) immunoblotting (IB) to examine the protein expression of MMP-9, TIMP-1 and COX-2. The representative bands in the IB from six rabbits in each group are shown. ^*^P<0.05 vs. Con group; ^+^P<0.05 vs. CRF group.

In order to examine whether exogenous MMP-9 treatment affected the endogenous expression of MMP-9, the renal mRNA and protein expression of MMP-9 following a four-week treatment period of MMP-9 in adenine-induced CRF rabbits was examined using qPCR (Fig. 4A) and immunoblotting (Fig. 4B), respectively. There was abundant MMP-9 expression of mRNA and protein in the control group; however, in the adenine-induced CRF group, a marked reduction in MMP-9 expression was observed (P<0.05). The low level of MMP-9 was significantly improved by exogenous MMP-9 treatment (P<0.05).

### MMP-9 decreases the expression of TIMP-1 and COX-2

It has been indicated that TIMP-1 may be positively correlated with renal function damage (11). In the current study, the serum level of TIMP-1 was significantly increased in adenine-induced CRF (P<0.05 versus the control, Fig. 1E). Thus, serum levels of TIMP-1 were examined to investigate whether the improvement in renal function due to MMP-9 was accompanied by decreased serum levels of TIMP-1. The results showed that MMP-9 treatment significantly decreased the serum level of TIMP-1 at the 11th week (the first week subsequent to MMP-9 treatment) compared with that in the CRF group (P<0.05, Fig. 2B). The serum level of TIMP-1 in the MMP-9-treated group increased gradually with time; however, it remained lower than that in the CRF group. By contrast, the serum level of TIMP-1 in the control and CRF groups remained virtually unchanged (data not shown). Following this, whether MMP-9 treatment affected the renal expression of TIMP-1 was examined using qPCR and immunoblotting. Consistent with the results from the serum, the renal mRNA and protein expression levels of TIMP-1 were increased in the CRF group compared with those in the control group. MMP-9 treatment significantly decreased TIMP-1 expression (P<0.05, Fig. 4B).

The expression of the proinflammatory factor COX-2 in the kidney was also examined. The COX-2 mRNA and protein expression levels were markedly increased in the CRF group compared with those in the control group (P<0.05); however, MMP-9 treatment significantly reduced the expression (P<0.05, Fig. 4).

## Discussion

As a key enzyme involved in ECM degradation, MMP-9 has been observed to be differentially expressed in acute and chronic renal disease models. In acute glomerulonephritis, MMP-9 expression increases, in parallel with the development of abnormal glomerular histology (18). However, in numerous CKD models, the expression and activity of MMPs have been shown to decrease (19,20). The decreased expression and activity of MMPs have been suggested to be associated with the development of tubulointerstitial fibrosis and glomerulosclerosis (19,20), in addition to the exacerbation of renal function (21). Consistent with these observations, our results in an adenine-induced model of CRF revealed decreased MMP-9 expression in the kidney, impaired renal function, proteinuria, tubulointerstitial fibrosis and glomerulosclerosis.

To date, there has been no direct and conclusive experimental evidence to support the presumed protective role of MMPs in CKD. The data in this study revealed that exogenous MMP-9 decreased the levels of SCr and BUN, reduced proteinuria and improved kidney morphology. Our data also revealed that exogenous MMP-9 treatment induced MMP-9 mRNA and protein expression in the kidney, which demonstrated that MMP-9 stimulated endogenous MMP-9 expression. The endogenous MMP-9 levels in the present study, including the serum level during adenine administration and the tissue level prior to or following MMP-9 treatment, were negatively correlated with the impairment of renal function and morphology. With regard to the involvement of exogenous MMP-9, the results of the present study have, to the best of our knowledge, for the first time, directly demonstrated the protective role of MMP-9 in CRF. Endogenous MMP-9 levels may be a useful marker for the evaluation of CRF. Moreover, MMP-7, another member of the MMP family, has been considered as a noninvasive biomarker of profibrotic signaling in obstructive nephropathy and focal segmental glomerulosclerosis (22).

In general, TIMP-1 shows contrasting changes to MMP-9 in CKD. The abundance of TIMP-1 in the kidneys has been shown to significantly increase in the majority of experimental models and several human renal diseases, showing positive correlation with the extent of fibrosis (23,24). The overexpression of TIMP-1 in a transgenic mice model promoted renal interstitial fibrosis through the inflammatory pathway, which may have been partly induced by the upregulation of intercellular adhesion molecule-1 (ICAM-1), a non-ECM substrate of MMPs (3). In the present study, the TIMP-1 levels in the serum and kidney increased during adenine-induction. While renal function and morphology were improved by MMP-9 treatment, the TIMP-1 level was significantly decreased. These results further demonstrated the positive correlation between TIMP-1 expression and renal damage. The decreased serum level and renal expression of TIMP-1 may mediate the protective effects of MMP-9 in CRF.

Following the initial reduction at the first week subsequent to MMP-9 treatment, the serum level of TIMP-1 gradually increased, although the renal function gradually improved. The inverse correlation between the serum level of TIMP-1 and renal function impairment was not consistent with the positive correlation mentioned previously. In this study, the injection of MMP-9 into the renal arteries notably increased the serum level of MMP-9, which was accompanied by rapid and marked decreases in the serum level of TIMP-1. Furthermore, the contrasting changes in TIMP-1 and MMP-9 levels were apparent during adenine administration. As the tissue inhibitors of MMPs, TIMPs bind to MMPs to form high-affinity complexes (7). It has been indicated that the high-affinity complexes of MMP-9 and TIMP-1 may contribute to TIMP-1 clearance. However, further investigation is required.

Inflammation is another important factor involved in CRF. An increased expression of COX-2 was observed in an adenine-induced model of CRF (15). Furthermore, celecoxib, a selective COX-2 inhibitor, was demonstrated to attenuate cisplatin-induced nephrotoxicity (25). In the present study, MMP-9 treatment significantly decreased adenine-stimulated COX-2 expression in the kidney, suggesting that an anti-inflammatory effect may be another action mechanism underlying the protective role of MMP-9 in CRF.

With the advance in biomedicine, the role of MMPs/TIMPs in various organs is becoming clear. The imbalance in the MMPs/TIMPs ratio is important in the early stage of osteoarthritis (26), bleomycin-induced pulmonary fibrosis (27) and liver fibrosis (28). A variety of treatments are being developed to improve fibrosis by interfering with the imbalance in MMPs/TIMPs, such as hepatocyte growth factor in liver fibrosis (12) and all-trans retinoic acid in glomerulosclerosis (29). With regard to the protective role of MMP-9 in CRF explored in the present study, investigating methods to increase MMP-9 levels is likely to be beneficial in the search for efficient therapies for tubulointerstitial fibrosis and glomerulosclerosis in CRF.

In conclusion, the results presented in this study demonstrate the protective role of MMP-9 in CRF. MMP-9 may exert the protective effect directly via its ability to degrade the ECM or through the suppression of its endogenous inhibitor, TIMP-1, and the proinflammatory response.

## Figures and Tables

**Table I:** Sequences of primers.

Primer	Sense (5′-3′)	Antisense (5′-3′)	Accession number
β-actin	GTGAGATGCCATGTGACGGA	TACACAAATGCGATGCTGCC	NM_001101683.1
MMP-9	GGGCTACGTGAGCTTTGACA	AAACTGGTCCCTTCCCCGTC	NM_001082203.1
TIMP-1	CCGGACAGACGCTAGAGAATC	AAGGTCGGAGTTGCAGAAGG	NM_001082232.2
COX-2	TGAACTTCCAAGCTGGCCTC	CCGATGCACAACTGAACTGG	NM_001082388.1

MMP-9, matrix metalloproteinase-9; TIMP-1, tissue inhibitor of metalloproteinase-1; COX-2, cyclooxygenase-2.
